# PARTICIPANTS’ PERCEPTIONS OF A COMMUNITY-BASED DANCE PROGRAM

**DOI:** 10.1093/geroni/igz038.515

**Published:** 2019-11-08

**Authors:** Megan R Bewernitz, Cari Freiberger

**Affiliations:** Jacksonville University, Jacksonville, Florida, United States

## Abstract

Parkinson’s disease (PD) is a progressive disease that targets many aspects of the nervous system, both motor and non-motor functions. Dance as an intervention provides a creative outlet that can be more motivating than other interventions by allowing people an opportunity for not only increased movement and activity, but also socialization. Creative expression through dance also has the potential to improve not only motor capabilities like balance, but also non-motor aspects like self-efficacy, cognition, and quality of life. For the community program described in this presentation, dance faculty led the dance and choreography components of the program while an occupational therapist (OT) served to maximize options for adaptations, safety, and accessibility. OT faculty also led efforts in gathering and analyzing data regarding community participation and program design utilizing a qualitative approach by conducting semi-structured interviews with five participants (four with PD, one with hydrocephalus, all over age 65) in the program. Content analysis of the data suggests some emerging themes which include: 1) forming common bonds and making friendships within the class due to PD diagnosis and similar enjoyment of the class, 2) increased appreciation and enjoyment of music, 3) appreciation of dance instruction, and 4) how transportation options, or lack thereof, impact participation in the class. These themes will assist in informing future program delivery with the hopes of reaching a larger contingent of the community who may also be interested in dance.

